# Septic Arthritis Caused by Kingella Kingae: A Case Report

**Published:** 2011-12-01

**Authors:** J Mardaneh, G Eslami, F Fallah, H Goudarzi, M M Soltan Dallal

**Affiliations:** 1 Department of Pathobiology, School of Public Health and Institue of Public Health Research, Tehran University of Medical Sciences, Tehran, Iran; 2 Prof. Alborzi Clinical Microbiology Research Center, Shiraz University of Medical Sciences, Shiraz, Iran; 3 Department of Microbiology, Shaheed Beheshti University of Medical Science, Tehran, Iran

**Keywords:** Septic arthritis, Kingella kingae, Iran

Dear Editor,

Kingella kingae, a short gram- negative rod, is part of the normal oropharyhgeal flora, but rarely, it has been implicated as a cause of clinical infection. In spite of significant differences between Moraxella sp. and Kingella kingae, it has been classified in the genus of Moraxella but recent studies have prompted placement in a separate genus of Kingella. Kingella kingae is an infrequent cause of septic arthritis, particularly during childhood.[1] In this report, we describe a child who had septic arthritis caused by this organism. The apparent rarity of documented case may be partly a manifestation of clinical unfamiliarity and the fastidious nature of the organism that made its identification difficult.

An 8-year-old by was hospitalized for fever, pain and swelling in knee joint. From some days before his admission, the child could not spontaneously move the right knee joint and his past medical history revealed a diagnosis of minor thalassemia when he was seven years old. Physical examination showed pain, swelling in right knee and high fever (38.5oC). Laboratory investigations showed a peripheral leukocyte count that was 7,700/ml. The erythrocyte sedimentation rate was 49 mm/h. The radiograph of knee was normal. A sample of slightly sanguineous and purulent fluid was withdrawned from the knee joint. Synovial fluid was transferred to trypticase soy broth and no organism was seen in the gram stained smears incubated in 37 oC during 24 hours. The prepared smear from TSB medium and gram stain revealed abundant short plump gram- negative rods that tended to occur in pairs and short chains. Subsequently, we observed pure growth on blood agar and chocolate agar of the organisms, which had gram stain characteristics identical to those observed on the initial TSB medium smear. The organism was eventually identified as Kingella kingae. In addition, the strain was also susceptible to β-lactam antibiotics. Blood culture obtained after joint aspirated was sterile. The strain was catalase negative and oxidase positive.The colonies were surrounded with narrow zone of β-hemolysis.

K. kingae was individualized by Henriksen and BØvre in 1976.[1] It was infrequently reported as a case of clinical infection. It has been isolated from blood, nose, skin lesions, and an abscess of the eyelid.[2] A literature search disclosed two cases reported of K. kingae arthritis. Joint fluid revealed K. kingae, however, in only one of these cases, a 13- year-old male with septic arthritis of the hip joint was noticed.[3] The same authors also reported two case of K. kingae osteomyelitis, two other reports reported Moraxella sp. The Center for Disease Control has accumulated 75 clinical isolates of K. kingae that 21 cases were from bone or joint regions.[2][4][5] The paucity of documented cases of septic arthritis may be more apparent than real and may reflect problems of recognition and identification.[6] There were five case reports of endocarditis caused by K. kingae too.[7] The initial diagnosis was not easy because of its difficulty to growth in blood culture.

The source of the organism in our patients, like to many other case reports, was uncertain but K. kingae was isolated from the nasopharynx as part of the normal flora.[8][9][10] Our patient did not have any symptoms in upper respiratory tract for presence of infection and one could speculate this infection resulted in K. kingae bacteremia. Antimicrobial susceptibility test was limited. The organism has been uniformly susceptible to penicillin, ampicillin, ampicillin, erythromycin, tetracycline, trimethoprim- sulfomethoxazxole and chloramphenicol.[3] Penicillin would appear to be antibiotic of choice.

The finding of a gram negative bacillus with rather typical morphology in clinical material should make one consider K. kingae in the identification process, such a awareness that may better define the role of the organism as a human pathogen. The limited data from the Center for Disease Control suggest that it may not be as uncommon as reflected by the few published reports.
